# Planarians (Platyhelminthes)—An Emerging Model Organism for Investigating Innate Immune Mechanisms

**DOI:** 10.3389/fcimb.2021.619081

**Published:** 2021-03-01

**Authors:** Luis Johnson Kangale, Didier Raoult, Pierre-Edouard Fournier, Prasad Abnave, Eric Ghigo

**Affiliations:** ^1^ Aix-Marseille Univ, IRD, AP-HM, SSA, VITROME, Marseille, France; ^2^ Institut Hospitalo-Universitaire-Méditerranée-Infection, Marseille, France; ^3^ Aix-Marseille Univ, IRD, AP-HM, MEPHI, Marseille, France; ^4^ Special Infectious Agents Unit, King Fahd Medical Research Center, King Abdulaziz University, Jeddah, Saudi Arabia; ^5^ Regional Centre for Biotechnology, Faridabad, India; ^6^ TechnoJouvence, Marseille, France

**Keywords:** Platyhelminth, planarian, antimicrobial, stem cells, innate immunity, stem cells, toll-like receptor

## Abstract

An organism responds to the invading pathogens such as bacteria, viruses, protozoans, and fungi by engaging innate and adaptive immune system, which functions by activating various signal transduction pathways. As invertebrate organisms (such as sponges, worms, cnidarians, molluscs, crustaceans, insects, and echinoderms) are devoid of an adaptive immune system, and their defense mechanisms solely rely on innate immune system components. Investigating the immune response in such organisms helps to elucidate the immune mechanisms that vertebrates have inherited or evolved from invertebrates. Planarians are non-parasitic invertebrates from the phylum Platyhelminthes and are being investigated for several decades for understanding the whole-body regeneration process. However, recent findings have emerged planarians as a useful model for studying innate immunity as they are resistant to a broad spectrum of bacteria. This review intends to highlight the research findings on various antimicrobial resistance genes, signaling pathways involved in innate immune recognition, immune-related memory and immune cells in planarian flatworms.

## Introduction

All animals are exposed to microbes present in their immediate environment and thus require protective mechanisms against infectious agents for their survival. All such fundamental interactions between hosts and pathogens are of considerable medical interest. The recent exPLoSion of knowledge on the evolutionary, genetic, and biochemical aspects of the interaction between the innate immune system and microbes has renewed scientific interest in exploring the invertebrate organisms (Pittenger et al., 1999; Tomchuck et al., 2008; Allam and Raftos, 2015; Milutinovic and Kurtz, 2016). Invertebrates are interesting organisms to study because they are relatively simple, lack an adaptive immune system, and have immune-competent cells, presenting a certain level of similarity with vertebrate phagocytes (Flannagan et al., 2012; Gold and Bruckner, 2015; Abnave et al., 2017; Hartenstein and Martinez, 2019). The ease of genetic tractability, an amenity to perform high-throughput screenings, and the absence of adaptive immunity make invertebrates a convenient model system for exploring conserved antimicrobial immune responses (Takeda et al., 2003; Buchmann, 2014; Myllymaki et al., 2014; Hillyer, 2016; Mangoni et al., 2016).

In invertebrates, the cellular immune response against microbes is associated with the presence of phagocytes or cells having immune function closely related to macrophages from vertebrates, so-called macrophage-like cells (e.g., amebocytes, hemocytes, and coelomocytes) (Ottaviani and Franceschi, 1997; Gold and Bruckner, 2015; Abnave et al., 2017). Like in the vertebrates, the recognition of microbes by invertebrates also involves the expression of pathogen recognition receptors (PRRs) by host immune cells, that recognizes pathogen-associated molecular patterns (PAMPs) expressed by microbes (Janeway and Medzhitov, 2002; Kumar et al., 2011; Gulati et al., 2018). The most described PRRs expressed on host cells are Toll-like receptors (TLRs) (Takeda et al., 2003), Nod-like receptors (NLRs) (Kim et al., 2016; Platnich and Muruve, 2019) and scavenger receptors (Peiser and Gordon, 2001; Pluddemann et al., 2007). The engagement of PRR through the binding of PAMP initiates a signal transduction cascade involving mitogen-activating protein kinases (MAPKs) and leads to complex cellular reactions. Indeed, the engagement of PRR activates phagocytosis and production of innate immune effectors, such as reactive oxygen intermediates, reactive nitrogen intermediates, antimicrobial peptides, lectins and cytokines, to eliminate the invader (Kawai and Akira, 2007; Kawasaki and Kawai, 2014; Satoh and Akira, 2016).

**Figure 1:**
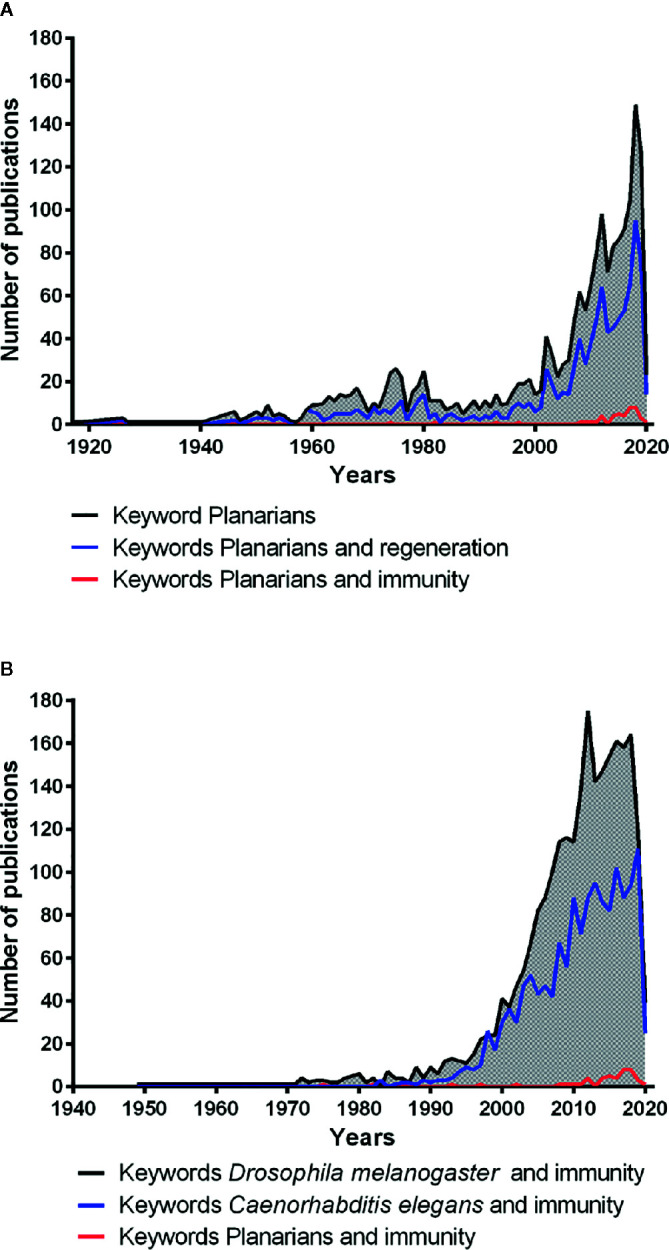
Number of publications related to the scientific field planarians (PubMed database). **(A)** number of publications related to planarian using the keywords planarians (black line); planarians and regeneration (blue line); planarians and immunity (red line). **(B)** number of publications related to the immunity in model organisms using the keywords *Drosophila melanogaster* and immunity (black line); *Caenorhabditis elegans* and immunity (blue line); planarians and immunity (red line).

The free-living planarian flatworms are invertebrates from the large phylum of Platyhelminthes. They live in freshwaters, such as lakes, ponds, and rivers, and have a worldwide distribution. Platyhelminthes probably have appeared on earth approximately 540–400 million years ago (Ruiz-Trillo et al., 1999). Planarians are zoophages (Klionsky et al., 2015) and have attracted considerable scientific attention (https://pubmed.ncbi.nlm.nih.gov/) (**Figure 1A**, black line) because of their extraordinary capacity to regenerate after amputation. This extraordinary regeneration capacity in planarians is due to the presence of a large number of adult pluripotent stem cells distributed throughout their body, except in the pharynx and the brain region (Ivankovic et al., 2019). These pluripotent stem cells possess the ability to give rise various types of linage committed stem cells which ultimately produce all different types of differentiated cell types in the planarian body (Wagner et al., 2011; Scimone et al., 2018). Shaw in 1790, Drapernauld in 1800, Dalyell in 1814, Johxson in 1822 and1825, Van Duyne in 1896, and Randolfh in 1897 showed that tissue fragments taken from almost any part of the planarian body could develop into an entire worm (Morgan, 1898). TH Morgan, in 1898, based on the observations and experiments made by his colleagues, re-investigated planarian regeneration and analyzed the different orientation planes of their regeneration. He concluded that planarians are immortal under the knife as they can survive after decapitation and are able to regenerate their head or generate a new body from the remaining piece of head (Morgan, 1898). Planarians had also attracted the attention of E. Metchnikoff. Indeed, in his work memories published in 1901, he described planarian use to investigate internalization and digestion of red blood cells by their intestinal cells as some of them are transparent and have larger size than protozoans (Metchnikoff, 1901). Since 1790, planarians are being used in various investigations, for example, as toxicology models (Fries, 1928), for locomotion investigations (Stringer, 1917), as grafting models (Rand and Browne, 1926), and as regeneration models (Morgan, 1898). These remained the major topic studied by using planarians, as evidenced by the scientific literature indexed under PubMed (**Figure 1A**, blue line). The scientific interest for the planarians renewed in approximately in the year 2002 and 2003, with a total of 64 scientific papers published because of the availability of new genetic tools (Salo and Baguna, 2002; Newmark et al., 2003), and the number of papers reached a maximum of 149 publications in 2018. Interestingly, planarians also have a remarkable ability to fight against large-spectrum of microbes (such as bacteria and fungi) that are known pathogens for humans. However, the immune capacity of planarians has been poorly investigated (**Figure 1A**, red line) as compared to the regeneration studies (**Figure 1A**, red line and blue line) and also in comparison with the immunity-related studies in *Drosophila melanogaster* (Insecta) and *Caenorhabditis elegans* (Secernentea) (**Figure 1B**). Previous studies have demonstrated that planarians can efficiently deal with several human pathogenic bacteria that also cause lethal infections to other invertebrates such as *D. melanogaster* and *C elegans* (Abnave et al., 2014). Hence planarians may provide us with a unique opportunity to investigate the antibacterial resistance mechanisms against human pathogens. Their finding may reveal some unknown molecules or unusual strategies to fight against human pathogens.

## Brief History of the Antimicrobial Response in Planarians

While investigating regeneration in the planarian *Dugesia dorotocephala via* electron microscopy, Morita et al. in 1974 observed that mesenchymal cells have phagocytosis capacity as they could internalize the cellular debris (Carpenter et al., 1974; Morita and Best, 1974). However, the link between this phagocytic capacity and immune activity against microbes could not been established. In 1991, Morita attempted to investigate this link by using *Mycobacterium tuberculosis*, the causative agent of tuberculosis. Heat-killed *M. tuberculosis* (strain H37Ra) was inserted in an incision made behind the eye of the planarian *D. dorotocephala*. The authors reported that reticular cells recognized the dead bacteria and engulfed them within a vacuole. This result suggested phagocytic activity is possibly related to the specific recognition of the microbes and revealed the presence of innate immune functions in planarians (Morita, 1991). However, no further studies explored or validated the immune mechanisms in planarians until very recently. More than 20 years later, the antimicrobial response of planarians was again investigated (Abnave et al., 2014) and numerous immunity related genes and proteins were identified (**Table 1**).

**Table 1:** Immune genes identified in planarians specie *S. mediterranea* and *D. japonica* to date.

*S. mediterranea*		*D. japonica*	
Names	References	Names	References
Smed-ARNTL	(Tsoumtsa et al., 2017)	Dj-TRAF3	(Pang et al., 2016)
Smed-cyld-1	(Arnold et al., 2016)	Dj-RIG-I	(Pang et al., 2016)
Smed-hep	(Arnold et al., 2016)	Dj-GRP78	(Ma et al., 2014)
Smed-jnk	(Arnold et al., 2016)	Dj-C-type lectin-like	(Gao et al., 2017)
Smed-jun D	(Arnold et al., 2016)	Dj-GILT	(Gao et al., 2020)
Smed-LTA4H	(Hamada et al., 2016)	Dj-14-3-3	(Lu et al., 2017)
Smed-mkk4	(Arnold et al., 2016)	Dj-Plac8	(Pang et al., 2017)
Smed-mkk6-1	(Arnold et al., 2016)	Dj-PLSCRs	(Han et al., 2017)
Smed-MyD88	(Tsoumtsa et al., 2018)		
Smed-p38	(Arnold et al., 2016; Torre et al., 2017; Tsoumtsa et al., 2017; Maciel et al., 2019)	Dj-p38	(Pang et al., 2016)
Smed-pgrp-1	(Arnold et al., 2016; Torre et al., 2017)		
Smed-pgrp-2	(Arnold et al., 2016; Torre et al., 2017)		
Smed-pgrp-3	(Arnold et al., 2016; Torre et al., 2017)		
Smed-pp6	(Arnold et al., 2016)		
Smed-ppm1a	(Arnold et al., 2016)		
Smed-ppm1b	(Arnold et al., 2016)		
Smed-setd8-1	(Torre et al., 2017)		
Smed-SRAM	(Tsoumtsa et al., 2018)		
Smed-tab1-1	(Arnold et al., 2016)		
Smed-tak1	(Arnold et al., 2016; Maciel et al., 2019)		
Smed-TIM	(Tsoumtsa et al., 2017)		
Smed-TIR	(Tsoumtsa et al., 2018)		
Smed-Traf2	(Arnold et al., 2016)	Dj-TRAF2	(Hu et al., 2019)
Smed-Traf2-1	(Arnold et al., 2016)		
Smed-Traf6	(Arnold et al., 2016; Tsoumtsa et al., 2017)	Dj-TRAF6	(Pang et al., 2016)
Smed-xiap	(Arnold et al., 2016)		
Smed-morn2	(Torre et al., 2017; Tsoumtsa et al., 2017; Maciel et al., 2019)	Dj-morn2	(Abnave et al., 2014; Tsoumtsa et al., 2017)
Smed-FoxF-1	(Scimone et al., 2018)		

## Antimicrobial Resistance Genes in Planarians

### Autophagy Genes

To investigate the innate immunity of planarians, Abnave et al. in the year 2014 (Abnave et al., 2014) infected *Dugesia japonica* by feeding them with 16 strains of human pathogenic bacteria, that are also lethal for model organisms, such as Drosophila and nematodes (**Supplementary Table 1**). The set of pathogens tested in this study included pneumophila (*Legionella pneumophila*), brucellosis (*Brucella melitensis*), salmonellosis (*Salmonella typhimurium*), listeriosis (*Listeria monocytogenes*) and tuberculosis (*Mycobacterium tuberculosis*). Authors observed that the 16 strains of bacteria were eliminated from the planarian body within few days. The researchers found that bacteria were eliminated by the process phagocytosis within gut cells. This capacity to cope with bacterial infection is not restricted to *D. japonica*, since the planarian species *Schmidtea mediterranea* was also able to eliminate the microbes tested in the study. Interestingly, increasing the temperature of planarian culture up to 28°C increases the capacity of planarians to cope with microorganisms (Hammoudi et al., 2018). To decipher the mechanism involved in this strong resistance against microbes, the researchers analyzed the *D. japonica* transcriptomic profile in response to bacterial infection by RNA sequencing. Among the 1400 genes modulated, 18 genes were identified through RNAi screening to be required for the robust elimination of the microbes tested. Further, the findings revealed that 8 genes out of the 18 genes are required to control the infection against gram-positive, gram-negative bacteria, and mycobacteria. Among those, authors focused on the gene MORN2. At the time of the study, the immunological function of MORN2 was utterly unknown. Surprisingly, the *D. japonica (DJ)-MORN2* has an orthologue in *Humans* (with ~48% identity) but is absent in the invertebrates such as *D. melanogaster* and *C. elegans*. While investigating the immunological function of *MORN2*, authors revealed that overexpression of *Hs-MORN2* in human macrophages promotes the elimination of bacteria, such as *L. pneumophila* or *M. tuberculosis*.

Analysis of the mechanism involved in the bacterial killing driven by *Hs-MORN2* indicates that *Hs-MORN2* induces the elimination of the bacteria in a phagolysosome by activation of LC3-associated phagocytosis (LAP), a type of autophagy (**Box 1**) (Klionsky et al., 2015; Heckmann and Green, 2019). While autophagy and LC3-associated phagocytosis are well studied in invertebrates, the LAP has not been described to be involved in microbial clearing (Fazeli et al., 2016; Kuo et al., 2018). Notably, transfection of *Dj-MORN2* into human macrophages had a similar effect to that observed during the overexpression of *Hs-MORN2* in human cells, suggesting a conserved antibacterial function of MORN2 protein. Mechanistically, it appeared that MORN2 interacts physically with LC3 and sequestosome-1 (SQSTM1/P62), promoting the conversion of LC3-I to LC3-II and its association with phagosome-containing microbes. LAP promoted by MORN2 requires Beclin-1, and Atg5 whereas it’s independent of components of the autophagy preinitiation complex (Ulk1, Atg13, and FIP2000) (Huang and Brumell, 2014). This work investigating the genetic and functional studies of host-bacterial interactions in planarians revealed the critical and conserved functions of innate immune components. The extraordinary capacity of planarians to cope with microbes has also been found during infection with *Candida albicans*, a fungal pathogen affecting humans (Maciel et al., 2019). *Candida albicans* is an opportunistic pathogen colonizing the gastrointestinal tract in over 50% of healthy individuals. It can cause severe and recurrent infections of the mucosa, as well as life-threatening systemic infections (Coronado-Castellote and Jimenez-Soriano, 2013; Mayer et al., 2013). Invertebrates, such as *Galleria mellonella*, the honeycomb moth, *D. melanogaster* and *C. elegans*, die rapidly after infection with *C. albicans* (Chamilos et al., 2006; Pukkila-Worley et al., 2009; Fuchs et al., 2010; Rossoni et al., 2017). In contrast, the protochordate amphioxus, can deal with *C. albicans via* the chitin-binding domain of the enzyme chitotriosidase (Xu and Zhang, 2012). Indeed, *C. albicans* infects and grows within planarians, inducing tissue damage. However, despite this infection, *S. mediterranea* rapidly clears *C. albicans*, and tissue damage is restored within a few days. The elimination of *C. albicans* by planarians is associated with the induction of *Smed-MORN2*, *Smed-TAK1* and *Smed-p38* genes (Maciel et al., 2019), demonstrating the importance of MORN2 in planarian immunity against fungal infections as well. A recent study published in the year 2020 by Morita et al., has validated the conserved function of murine MORN2 in LAP mediated killing of *E. coli* in murine macrophages (Morita et al., 2020).

Box 1 **Phagocytosis and Autophagy.**

**Phagocytosis** consists of the uptake and digestion of particles, including microbes. Microbes are internalized in phagosomes, which then mature and fuse with lysosomes to form degradative phagolysosomes.

**Autophagy** is the selective engulfment of the cytoplasm, which can contribute to antibacterial immunity. Bacteria that escape or damage phagosomal membranes are recaptured by the autophagic system, which consists of double-membrane compartments that fuse with lysosomes. Autophagy is defined by its dependence on components of the autophagy preinitiation complex formed by Ulk1, Atg13, and FIP2000 and is structurally defined by the presence of double-membrane compartments.

**LC3-Associated phagocytosis LAP:** Bacterial recognition links components of the autophagy pathway to phagocytosis through a mechanism called LC3-associated phagocytosis (LAP). LAP genetically requires Beclin1, Atg5, and Atg7 and is structurally defined by the presence of single-membrane compartments.

### Glucose-Regulated Protein 78 Gene

Following the work done by Abnave et al. on planarian innate immunity (Abnave et al., 2014), several studies exploring planarian immunity have appeared in the literature. Ma et al. characterized the *Dj-GRP78* (78 kDa glucose-regulated protein) from *D. japonica* (Ma et al., 2014). Dj-GRP78 shows a 76.4% similarity with GRP78 from the nematodes *C. elegans* and *Homo sapiens*. In mammals, GRP78 (BiP/HSPA5) functions as a heat shock protein and is induced during oxidative stress (Nakajima and Kitamura, 2013). In the vertebrates, it has been suggested that GRP78 expression protects the host cell against the accumulation of superoxide (Nakajima and Kitamura, 2013). It has also been reported that the inhibition of GRP78 induces the expression of MCP-1 and promotes macrophage infiltration (Cook et al., 2016). In sea urchins (Falugi et al., 2012) and shrimps (*Fenneropenaeus chinensis*, *Penaeus monodon*) (Luan et al., 2009; Li et al., 2018), the expression of *GRP78* is associated with a response to stress (i.e., pH, heavy metal). Moreover, in *C. elegans*, GRP78 protein expression is related to the expression of antimicrobial peptides required to control nematode infection with the fungal pathogen *Drechmeria coniospora* (Wang et al., 2015). Similarly, GRP78 is implicated in the immune response of *Apostichopus japonicus* against *Vibrio splendidus* (Lamitina and Chevet, 2012). Planarians challenged with *Escherichia coli* (non-pathogenic laboratory strain DH5α) by co-incubation with an amputated tissue showed enhanced expression of *Dj-GRP78*, suggesting the possible role of *Dj-GRP78* in planarian innate immunity. Further studies are needed to explore the immune pathways engaging GRP78 in planarians during infections (Ma et al., 2014).

### Leukotriene A4 Hydrolase Gene

LTA4H is a hydrolase that plays a role in the restriction of bacterial growth in vertebrates. LTA4H catalyses the last step of the synthesis of leukotriene B4 (LTB4), a potent pro-inflammatory lipid mediator derived from arachidonic acid (Snelgrove, 2011). LTA4H is a regulator controlling the balance of pro-inflammatory and anti-inflammatory eicosanoids, and the production of pro-inflammatory cytokines (i.e., TNFα). The inhibition of LTA4H reduces the lipopolysaccharide (LPS)-induced production of pro-inflammatory cytokines and promotes the production of cytokines, such as interleukin (IL)-10, thereby enhancing bacterial invasion or bacterial susceptibility (Tobin et al., 2010; Curtis et al., 2011; Snelgrove, 2011; Tobin et al., 2012; Yang et al., 2014; Dunstan et al., 2015). The analysis of the publicly available transcriptome revealed the existence of *S. mediterranea (Smed)-LTA4H*, an ortholog to *Hs-LTA4H* (44% similarity at the protein level) (Hamada et al., 2016). Planarian *S. mediterranea* is capable of clearing the *Staphylococcus aureus* infection, that can cause nosocomial disease, pneumonia, abscess, sepsis, and toxic shock syndrome (Abnave et al., 2014). However, silencing of the *Smed-LTA4H* gene by RNA interference promotes the *S. aureus* clearance in 4 days. In contrast, in vertebrates, *LTA4H* deficiency likely leads to bacterial proliferation and a failure to resolve the bacterial infection (Tobin et al., 2010; Curtis et al., 2011; Snelgrove, 2011; Tobin et al., 2012; Yang et al., 2014; Dunstan et al., 2015). It has been shown that inhibition of *LTA4H* expression by maresin-1 induces rapid regeneration of the planarian system (Serhan et al., 2012). In planarians, tissue regeneration requires autophagy (Gonzalez-Estevez et al., 2007), and autophagy is also one of the way for innate immunity to clear bacterial infection (Abnave et al., 2014; Schille et al., 2018). Thus, it may be hypothesized that in planarians, the silencing of the *Smed-LTA4H* gene could result in an increase in autophagy and a disruption of the tissue homeostasis equilibrium (controlled *via* autophagy), thereby leading to rapid elimination of the bacteria.

### Circadian Clock Genes

Both in the vertebrates and invertebrates, numerous biological mechanisms, including the immune system, are closely linked to the circadian rhythm (Tsoumtsa et al., 2016; Scheiermann et al., 2018). Clock control genes (CCGs) interact by forming posttranscriptional and posttranslational regulatory loops, creating a 24-h oscillation. In the main loop, *Clock*/*Bmal-1* promotes *Per* and *Cry* transcription during the daytime. PER and CRY are synthesized in the cytoplasm, dimerize and translocate in the nucleus, where they accumulate (Curtis et al., 2014). Fluctuations in the expression of clock genes lead to variation in the expression of several immune-related genes (*Stat-3, Stat-5, Erg-1, NF-kB*, and *Tlr-9*) which, in turn, may impact the capacity of immune system responsiveness towards aggression (Logan and Sarkar, 2012; Silver et al., 2012). Mice with *aryl hydrocarbon receptor nuclear translocator-like* (*Arntl*)*-1* knocked down are more susceptible to *Listeria monocytogenes* infection (Nguyen et al., 2013) than a wild-type mice. In invertebrates, such as *D. melanogaster*, deletion of the clock gene *Period-2* (*Per*)*-2* increases their susceptibility to *Pseudomonas aeruginosa, Streptococcus pneumoniae*, and *L. monocytogenes*. The elimination of the clock genes *circadian locomotor output cycles kaput* (*Clock*), *Cycle* (*Cyc*, a homologue of *Hs-Arntl-1*), and *Timeless (Tim)* causes an increase in resistance to *Pseudomonas aeruginosa* (Shirasu-Hiza et al., 2007; Lee and Edery, 2008). So far, no clear orthologue for Hs-*Clock* and Hs-*Per-2* has been discovered in planarian *S. mediterranea*. However, orthologues to *Hs-Tim* and *Hs-Arntl-1* have been identified (Tsoumtsa et al., 2017). In contrast to *Smed-Arntl-1*, the silencing of *Smed-Tim* is required for an efficient antibacterial immune response against *S. aureus* in *S. mediterranea* under 12 h/12 h light/dark cycle conditions. The *Smed-Tim* silencing affects the expression of antimicrobial gene responses, such as p38 MAP-Kinase and Traf6, which transduce signals of pathogen perception (Arnold et al., 2016; Pang et al., 2016), and morn2, which controls the LC3-associated phagocytosis of bacteria for their destruction (Abnave et al., 2014). Thus, *Smed-Tim* regulates the capacity of planarians to kill *S. aureus* by modulating the expression of antimicrobial genes (Tsoumtsa et al., 2017).

### C-Type Lectin

C-type lectins (CTLs) are a large superfamily of over 1000 proteins that contains one or more characteristic C-type lectin-like domains (CTLDs). CTLs are involved in several physiological processes such as development, homeostasis and immunity. In mammals, CTLs can be found as transmembrane proteins or as soluble secreted molecules. CTLs are well known pattern recognition receptors that can recognize both endogenous and exogenous ligands. Various cells from innate and adaptive immune systems express CTLs. Individual CTLs are expressed in the cell type specific manner, hence can serve as a marker for that specific cell type. It can recognize all different types of pathogens like bacteria, fungi, viruses and parasites. The pathogen recognition triggers an intracellular signaling cascade that induce a wide range of cellular and immunological responses essential for antimicrobial immunity (Brown et al., 2018). Proteins containing C-type lectin-like domains are also found in different planarian species. Several CTLs are differentially expressed in various tissues (such as mucus glands, body edge, etc.) of planarian *Girardia tigrina* (Shagin et al., 2002). However, their involvement in planarian innate immunity is unexplored. Recently, a novel C-type lectin-like protein is identified and characterized in planarian *D. japonica* (Gao et al., 2017). This *Dj-CTL* is expressed in the pharyngeal and epidermis and is up-regulated upon the induction of lipopolysaccharide, peptidoglycan, Gram-positive and Gram-negative bacteria, indicating that it may be involved in the immune responses. In regenerating worms, the CTL was found to be mainly expressed in early blastemas and its knockdown by RNAi slowed down the wound healing process during regeneration. These observations suggest that planarian CTL might be involved both in innate immune response as well as regeneration.

### Gamma-Interferon-Inducible Lysosomal Thiol Reductase

As name suggests, the GILT is a lysosomal enzyme that can reduce protein disulfide bonds. GILT is constitutively expressed in antigen-presenting cells in mammals and is involved in antigen presentation as well as bacteria invasion. The enzymatic activity of GILT facilitates the complete unfolding of proteins that are destined for lysosomal degradation. Planarian GILT also exhibit the thiol reductase activity (Gao et al., 2020). *Dj-GILT* is expressed in intestinal phagocytes in planarians and is overexpressed when worms are infected with LPS, E. coli, and V. anguillarum, suggesting that it might be involved in the immune response to bacterial infections. It’s been demonstrated that planarian GILT acts as an antioxidant and thus facilitates the clearance of Gram-negative bacteria by regulating H2O2 levels (Gao et al., 2020). Moreover, silencing of GILT results in delayed bacterial elimination by planarians, suggesting that it may also play a potential role in removing invasive bacteria.

### TNF Receptor-Associated Factors

Tumor necrosis factor receptor-associated factors (TRAFs) are the signal transducers for the TNF receptor superfamily that plays an important role in both adaptive and innate immunity. The major downstream signaling events mediated by TRAFs include activation of the transcription factor nuclear factor κB (NF-κB) and the mitogen-activated protein kinases (MAPKs) (Shi and Sun, 2018). So far seven TRAFs are reported in mammals of which six are typical members (TRAF1–6) and an atypical member (TRAF7). TRAFs are involved in the regulation of innate immunity, inflammation, cell proliferation, apoptosis, stress and antiviral responses. TRAFs have also been reported in invertebrates such as *D. melanogaster* (Liu et al., 1999; Grech et al., 2000; Zapata et al., 2000), *C. elegans* (Wajant et al., 1998), *Dictyostelium discoideum* (Wajant et al., 1998), and Planarian *D. japonica* (Hu et al., 2019). Planarian *Dj-TRAF2* is mainly expressed in the pharynx of an intact animals. Their expression goes up in in response to pathogen-associated molecular patterns such as LPS, PGN, β-Glu and Poly(I:C). Hinting that planarian TRAF2 may have a role in an innate immune response. TRAF2 is found to be localized in the cytoplasm, suggesting that it might function as a signal adapter in the cytoplasm of cells. However, the detailed mechanistic insights of its immunological function are yet to be explored.

### Fox-Family Transcription Factor

Forkhead-box-family transcription factor (foxF-1) is a member of the Forkhead-box (FOX) gene family. Forkhead gene was first identified in the *D. melanogaster*. The gene was found to be essential for normal gut development and its absence leads to “forked head” appearance. Later, several Forkhead related genes were discovered in several other invertebrate and vertebrate animals. So far 44 Fox genes are reported in human and mouse, 11 in Drosophila, 15 in *C. elegans*, and 45 in Xenopus (Golson and Kaestner, 2016). These transcription factors control wide variety of biological functions in the development and homeostasis of various cells and tissues, including immune cells. Planarians possess a foxF-1 gene that encodes a homolog of *Drosophila* Biniou and vertebrate FoxF (Scimone et al., 2018). It acts as a master transcriptional regulator of all non-body wall muscle cells in planarians. Planarian S*med-foxF-1* drives the regulatory program for muscle cell subsets as well as intestinal phagocytic cells that are capable of phagocytosing *E. coli*. Mammalian foxF-1 is also known to be involved in the regulation of pulmonary and gut development (Golson and Kaestner, 2016). In *C. elegans*, the FoxF/FoxC transcription factor LET-381, is shown to be required for the formation of coelomocytes (Amin et al., 2010), which are scavenger cells in the animal pseudocoelom cavity. These findings suggest that foxF-1 may very well be involved in innate immune response by regulating the development of such phagocytic cells.

### 14-3-3 Proteins Family

14-3-3 proteins are a family of conserved multifunctional proteins regulating various developmental processes. They act as regulator molecule and interact with different kinases as well as phosphatases. 14-3-3 proteins are regarded as a major class of molecular chaperones and with the potential to interact with over 200 proteins (including defense related proteins), the 14-3-3 proteins can regulate the signal transduction and impact the cell fate. 14-3-3 proteins play roles in multiple signaling pathways, including those controlling metabolism, hormone signaling, cell division and host defense. In Huh7 cells, a member of the 14-3-3 family, 14-3-3η is shown to interact with MDA5 and promote the MDA5-dependent IFNβ induction pathway by reducing the immunostimulatory potential of viral dsRNA within MDA5 activation signaling pathway (Lin et al., 2019). 14-3-3 also functions in regulation of host-pathogen interactions in plants (Lozano-Duran and Robatzek, 2015). Thus far, two members of the 14-3-3 family proteins, 14-3-3 α and ζ are reported in planarian *D. japonica* (Lu et al., 2017). Dj-14-3-3 α and ζ are predominantly expressed in the pharynx of worms. They are up-regulated in response to pathogen-associated molecular patterns like LPS, PGN, β-Glu and Poly (I:C). Their expression goes up within 1–5 h after exposure. Therefore, it can be speculated that they may participate in the early immune responses. Further molecular studies need to performed to validate the role of 14-3-3 α and ζ in planarian immunity.

### Placenta Specific Protein 8

Placenta specific protein 8 (Plac8) is also known as C15 protein or Onzin. In normal human T-lymphocytes, overexpression of Plac8 induces apoptosis (Mourtada-Maarabouni et al., 2013). Several reports have demonstrated the innate immune properties of Plac8 and it’s known to be involved in enhancing bactericidal activities of phagocytes, macrophages, and neutrophils (Ledford et al., 2007); in mediating CD4 T cell clones to clear genital tract infections (Jayarapu et al., 2010); in influencing the production of proinflammatory cytokines interleukin 6 (IL-6) (Allan, 2012) *etc*…. Planarian *D. japonica* (Dj-)plac8 gene is highly expressed in the pharynx and its protein can be found in the intestine and epidermis as well (Pang et al., 2017). As pharynx and epidermis are most likely to be interacting with the external environments and pathogens, it can be speculated that Plac8 may be associated with planarian immune response. Moreover, plac8 expression is also modulated in response to LPS and is peaked at 5 h post exposure. The authors have also performed the bacteriostatic tests of the purified planarian Plac8 at 5 μg and 10 μg concentrations. Planarian Plac8 clearly inhibited the growth of *E. coli* and *P. aeruginosa* in a dose-dependent manner (Pang et al., 2017). Moreover, planarian plac8 was also found to be involved in the process of the pharynx development and regeneration. Hence, planarian plac8 can be considered as a multifunctional gene with crucial roles in immune responses and development in planarians.

#### Phospholipid Scramblases

Phospholipid scramblases (PLSCRs) constitute a family of five homologous (PLSCR1–PLSCR5), lipid-raft-associated plasma membrane proteins, which are involved in the translocation of phospholipid between membrane leaflets (Han et al., 2017). These are conserved proteins that are found in all eukaryotic organisms. In mammals, these proteins play essential roles in various physiological processes, especially in the immune responses. In mice, the PLSCR1 expression gets increased in response to the stimulation with LPS, zymosan and turpentine (Lu et al., 2007). The overexpression of PLSCR1 was able to protect the lung epithelial cells from infection of *Staphylococcus aureus* α-toxin (Lizak and Yarovinsky, 2012). Moreover, PLSCR1 could suppress vesicular stomatitis virus proliferation by enhancing the IFN response and increasing expression of antiviral genes (Dong et al., 2004). These results indicate that PLSCR plays an essential role in the immune responses in mammals. However, PLSCRs are not well studied in the invertebrate organisms. A PLSCR gene is identified in the planarian *D. japonica* (Dj-PLSCR) (Han et al., 2017). It is mainly expressed in the pharynx of the intact and regenerating animals. Authors have observed increase in the expression of PLSCR in response to stimuli with the different pathogen-associated molecular patterns such as poly(I:C), LPS, PGN and β-Glu. This report suggests that PLSCR could be possibly involved in planarian immune response but further investigations are required validate its immunological function and mode of action.

## Signaling Pathways

### Retinoic Acid Inducible Gene-I Signaling Pathway

The recognition of microbes involves PRR and their related receptor signaling pathways. Retinoic acid-inducible gene (RIG)-I is a member of PRRs and plays a pivotal role in the immune response by recognizing and binding nucleic acids from viruses (Cui et al., 2008; Yoneyama and Fujita, 2009). The RIG-I signaling pathway involves tumor necrosis factor receptor-associated factor (TRAF)-6, TRAF3, and MAPK-p38 TRAF (Bradley and Pober, 2001). The RIG-I pathway is present both in vertebrates and invertebrates. Indeed, this pathway exists in *D. melanogaster* (along with Toll-like, IMD and JAK/STAT signaling pathways) (Leclerc and Reichhart, 2004; Baeg et al., 2005; Ferrandon et al., 2007), *C. elegans* (along with DBL, DAF-2/DAF-16, MAPK, Toll-like signaling pathways) (Pujol et al., 2001; Morita et al., 2002; Young and Dillin, 2004; Kwon et al., 2010), and in crustaceans (along with Toll-like and IMD signaling pathways) (Lan et al., 2013; Li and Xiang, 2013). Analysis of transcriptomic data from *D. japonica* identified four genes, *Dj-RIG-I*, *Dj-TRAF3*, *Dj-TRAF6* and *Dj-p38*, from the RIG-I-like receptor signaling pathway. The measurement of the levels of *Dj-RIG-I*, *Dj-TRAF3*, *Dj-TRAF6*, and *Dj-p38* expression in response to stimulation with pathogen antigens, such as LPS and peptidoglycan (PGN), suggest that they are likely to be involved in the planarian immune response (Pang et al., 2016). Similar results have also been found using the oyster *Crassostrea gigas*. Indeed, the expression of *C. gigas* (Cg)-RIG-1 increases in response to PAMPs such as poly(I:C), LPS, PGN, heat-killed *L. monocytogenes* and *Vibrio alginolyticus* (Zhang et al., 2014). Till date, the RIG-I pathway has been reported to be only associated with the antiviral innate immune response (Mao et al., 1996; Conesa et al., 2005; Feng et al., 2011; Nie et al., 2015).

While investigating planarian microbiota, While investigating planarian microbiota, Arnold et al. have reported that planarians possess a wide variety of bacterial species in their microbiota and their bacterial composition can severely affect the regeneration in planarians (Arnold et al., 2016). Authors found high Bacteroidetes to Proteobacteria ratio in their healthy planarian culture. The shift in the bacterial composition (such as the expansion of Proteobacteria) was observed in the poorly managed static planarian cultures, which led to the development of dorsal lesions, tissue degeneration, and lysis in planarians. This suggest that pathogenic shift of microbiota composition can adversely affect the tissue homeostasis and regeneration in planarians. By performing RNAseq analyses, Arnold et al. identified a series of innate immune components in planarians and noticed that Proteobacteria expansion coincides with the induction of several innate immunity genes (Arnold et al., 2016). The researchers performed a candidate gene RNAi screen to uncover tissue degeneration mediators in response to infection with the pathogenic bacteria found in planarian microbiota.

Authors focused on conserved members of the mammalian inflammatory signaling cascade and the infection-responsive IMD pathway from *D. melanogaster* (Myllymaki et al., 2014). The researchers identified several orthologues of either *H. sapiens* or *D. melanogaster* genes and categorized them in mediating (activators) or reducing (inhibitors) signal transduction (Kopp et al., 1999; Ferrandon et al., 2007; Mogensen, 2009; Dai et al., 2012; Xie, 2013; Herrington and Nibbs, 2016). Many of these genes showed modulated expression (**Supplementary Table 2**) in response to amputation in the presence of infection with Pseudomonas (Arnold et al., 2016). Besides, the RNAi screen revealed that TAK1 innate immune signaling module (TAK1/MKK/MAPK-p38) is responsible for compromised tissue homeostasis and regeneration during infection. Although the exact implication of these genes in the innate immunity of planarians remains to be explored, this study revealed the *S. mediterranea* genes from an evolutionarily conserved inflammatory signaling module and also highlighted their involvement in a complex tissue regeneration process in the presence of infection. These findings revalidate that there is a strong correlation between microbiota, innate immune system and regeneration.

### Toll-Like Receptor Signaling Pathway

TLRs play a vital role in initiating both innate and adaptive immune responses and are the most famous among all PRR receptors (Kawai and Akira, 2011; Moresco et al., 2011; Kawasaki and Kawai, 2014). The structure of TLRs is well characterized. TLRs are formed by an extracellular region containing leucine-rich repeats (LRRs) required for the sensing of PAMPs followed by a transmembrane region and an intracellular region containing a Toll/IL1R receptor (TIR) domain, responsible for signal transduction (O’Neill and Bowie, 2007). Five adaptors are known for Toll/IL-1R, namely, myeloid differentiation primary response 88 (MyD88), MyD88 adaptor-like (MAL), also known as toll-interleukin receptor adaptor proteins (TIRAP), TIR domain-containing adaptor protein inducing interferon β (TRIF), TRIF-related adaptor molecule (TRAM), sterile alpha and TIR motif-containing protein (SARM) (O’Neill and Bowie, 2007; Nilsen et al., 2015). Studies suggest that organisms, such as Rotifera and Platyhelminthes (including planarians), does not harbor TLR orthologues, whereas TLRs are widely conserved across several other animal species (Leulier and Lemaitre, 2008; Coscia and Giacomelli, 2011; Peiris et al., 2014; Gerdol et al., 2017). Even if functional TLRs are not present, the TIR domain-containing proteins do exist in these organisms (Peiris et al., 2014; Gerdol et al., 2017). TLR2 in *Nematostella vectensis* (Cnidaria) consists only of TIR domains and a transmembrane region whereas it does not contain an extracellular portion (Song et al., 2012). However, *N. vectensis* TLR is involved in response to pathogens by its interaction with other LRR-containing proteins. A similar organization was found in *Hydra magnipapillata* (Cnidaria), where PAMP detection and antimicrobial peptide expression are possible through the communication of HvTRR1 and HvTRR2 with HvLRR1 (Bosch et al., 2009). The investigations on the antimicrobial response in planarians have revealed the existence of several genes contributing to their antimicrobial defense, including *Morn-2*, *Traf3*, *Traf6*, *p38-MAPKinase*, *Erk*, *Jnk*, *Mkk4*, *Mkk6*, *Tim*, *Tak1*, *Tab1*, *JunD*, *Rig-1*, *PGRPs*, *Hep*, *SetD8*, and *LTA4H* (Abnave et al., 2014; Arnold et al., 2016; Hamada et al., 2016; Pang et al., 2016; Torre et al., 2017; Tsoumtsa et al., 2018). Most of them are orthologues of mammalian Toll-like receptor (TLR) signaling pathway components, such as *Traf3*, *Traf6*, *p38-MAPKinase*, *Erk*, *Jnk*, *Mkk4*, *Mkk6*, *Tak1*, and *Tab1* (Song et al., 2012; De Nardo, 2015), suggesting at least partial conservation of TLR signaling pathways in planarians. Previous studies have identified three TIR domain-containing proteins in planarians (Peiris et al., 2014; Gerdol et al., 2017). A recent study employing *in silico* analysis of 32,615 protein sequences from the planarian species *S. mediterranea* revealed a total of 20 protein sequences containing at least one TIR domain (Smed-TIR). A detailed analysis of the identified Smed-TIR did not allow the detection of any LRR or any immunoglobulin-like domain linked to a TIR domain. Besides, these data suggest that there is no IL1R orthologue in planarians. The study also revealed the presence of SRAM (Smed-SRAM) domain and a MyD88 (Smed-MyD88) domain in two separate gene sequences. It is important to note that SARM is the most ancient TLR adaptor predicted to be conserved in all living organisms. Interestingly, Smed-MyD88 is an orthologue to bivalve MyD88, but it does not cluster with the sequences identified in the phylogenetic analysis. Similarly, *Hydra magnipapillata* MyD88 is not phylogenetically associated with other MyD88, suggesting a different acquisition and evolution history of MyD88 in the animal kingdom. The immune function of MyD88 is ancient, and it has been demonstrated that the innate immune response of sea sponge *Suberites domuncula* (Porifera) against bacteria relies on a MyD88-dependent signaling pathway (Wiens et al., 2005). The studies have hypothesized that Smed-MyD88 contributed to the recognition of bacterial PAMPs (Janeway and Medzhitov, 2002; Kumar et al., 2011). Even though none of the studies on planarians has identified functional TLR in the form that has been described to date, the sequences that can substitute for TLR functions have been suggested. Further investigations are needed to functionally characterize the identified TIR domain-containing sequences in the antimicrobial response of planarians.

## Immune Related Memory in Planarians

The existence of innate immune memory in vertebrates and invertebrates, known as trained immunity, has been reported and defined as heightened immune responses against previously encountered pathogens (Milutinovic and Kurtz, 2016; Netea et al., 2016). The mechanism of trained immunity in vertebrates involves epigenetic reprogramming through histone modifications, particularly by histone methyltransferases, that modulate the expression of antimicrobial genes during re-infection (Netea et al., 2016; Pereira et al., 2016). Instructed immunity or trained immunity has been observed in several invertebrates in response to bacteria but also cestode. Indeed, immune memory is developed in *Periplaneta Americana* (Insecta) in response to *Pseudomonas aeruginosa* infection (Faulhaber and Karp, 1992), in *D. melanogaster* against *Streptococcus pneumoniae* infection (Cooper, 1968), and in *Macrocyclops albidus* (Crustacean) against the cestode *Schistocephalus solidus* infection (Kurtz and Franz, 2003). However, in contrast to vertebrates, the mechanism of trained immunity in invertebrates has not been elucidated in detail.

Planarians have also been explored for the presence of trained immunity. It’s been demonstrated that planarians can initiate a genetic program of instructed immunity against *S. aureus* re-infection that allows increased expression of specific antimicrobial genes that aid to clear the pathogen more rapidly. This immune memory function is independent of phagocytic cells, unlike in *D. melanogaster* intestinal immunity, but it requires neoblasts (stem cells). The trained immunity mechanism in planarians requires neoblasts as cellular actors and the expression of the *Smed-PGRP-2* peptidoglycan receptor and the *Smed-setd8-1* histone methyltransferase as molecular effectors. The signaling pathway also involves the genes *Smed-p38 MAPK* and *Smed-morn2*, which display facilitated expression associated with enhanced bacterial clearance. *Smed-PGRP-2* controls the induction of *Smed-setd8-1* and their downstream histone lysine methylation activity in neoblasts (Torre et al., 2017). The study has revealed an unusual mechanism underlying the planarian immune memory, and it would be interesting to check whether other invertebrates rely on a similar immune memory mechanism.

## Immune Cells in Planarians

The immune cells involved in planarian innate immunity remain poorly investigated. To date, three types of cells have been suggested that could contribute to planarian immunity: these are reticular cells, intestinal phagocytes, and neoblasts. It has been shown that reticular cells are able to internalize dead microbes, such as dead *M. tuberculosis* (Morita, 1991), but no further investigations have been done on reticular cells. The absorptive phagocytes are one the major cell types in the intestinal tract of the planarians (Forsthoefel et al., 2012; Scimone et al., 2018). These cells may possibly express pathogen recognition receptors like PGRPs and several other innate immune-related genes that are expressed in the intestinal tract of planarians (Abnave et al., 2014; Arnold et al., 2016; Torre et al., 2017; Scimone et al., 2018; Gao et al., 2020). Several genes expressed by intestinal phagocytes that are essential for their maintenance have been identified in the *S. mediterranea* (Forsthoefel et al., 2012). However, their implication and contribution in innate immunity remained unknown, since their genetic invalidation neither affects the planarian antibacterial response nor the trained immunity (Torre et al., 2017). Apart from these cells, the presence of other dedicated immune cells in planarians is still not reported. In other invertebrates, such as *D. melanogaster* (Gold and Bruckner, 2015), *Pediculus humanus* (Kim et al., 2011; Coulaud et al., 2014), and crustaceans to marine bivalves, the implication of phagocytic cells in their defense against microbes has been established (Abnave et al., 2017). It appears that in *S. mediterranea*, neoblasts (stem cells) play a central role in the innate immune memory (Torre et al., 2017). The role of stem cells in the invertebrate immunity remains poorly investigated. There is little evidence of the relationship between *Drosophila melanogaster* stem cells and microbes. In most cases, stem cells respond to microbes by producing various factors that initiate the activation of several types of effector cells (enterocyte muscle cells, enteroblasts), including hemocytes, which are required for the elimination of microbes (Bonfini et al., 2016). In contrast, stem cells play a crucial role in the immunity of vertebrates. They are equipped with PRRs (including TLRs), they possess both immunosuppressive and inflammatory properties, as well as harbor antibacterial capabilities (Pevsner-Fischer et al., 2007; Liotta et al., 2008; Krasnodembskaya et al., 2010; Kim et al., 2011; Machado Cde et al., 2013; Hamada et al., 2018). The planarian epidermis, pharynx, excretory glands/cells could be other possible tissues/organs that should be further investigated for their active participation in the antimicrobial defense.

## Concluding Remarks

Because of their relative simplicity, and availability of the molecular and genetic tools, invertebrate models, such as sponges (i.e., bath sponge), worms (i.e., platyhelminthes, annelids, nematodes), cnidarians (i.e., jelly fish), molluscs (i.e., bivalves and snail), crustaceans (i.e., crabs, shrimps), insects (i.e., flies, mites), arachnids (spider), echinoderms (i.e., sea stars, urchins), platyhelminthes (i.e., planarians), are being useful to unravel immune mechanisms that vertebrates have inherited from invertebrates. Therefore, recently there is a considerable interest among researchers in developing and the studying less regarded invertebrate organisms that may contribute a different perspective to our present understanding of complex host-pathogen interactions. Planarian is one such model system that could be used for antimicrobial response investigations. They are quite affordable and relatively easy to culture in the lab. We can quickly produce a large number of clonal populations of these animals by repetitive amputation and regeneration cycles. We can infect a large number of animals simply by feeding the bacteria mixed with their food. Infections can also be performed conveniently by injecting pathogens directly inside the gut or at any other desired locations. Planarians possess primitive innate immune system with relatively less complexity. Moreover, numerous immune-related genes within planarians have been identified recently (**Table 1**). All these features make planarians a convenient and exciting model to explore innate immune mechanisms. Planarians need to be future studied to investigate the signaling pathways engaged during bacterial infection, and also to identify the different cell types contributing to the immunity in planarians.

## Author Contributions

LK wrote the draft and contributed to the final version manuscript. DR, P-EF, PA, and EG corrected and wrote the manuscript. All authors contributed to the article and approved the submitted version.

## Conflict of Interest

EG was employed by TechnoJouvence.

The remaining authors declare that the research was conducted in the absence of any commercial or financial relationships that could be construed as a potential conflict of interest.

## Supplementary Material

Supplementary Table 1 List of the 16 bacterial species used in the Abnave et al. study (Abnave et al., 2014). The three main classes of pathogens responsible for human diseases and pathogens of model organisms, such as *C. elegans* or *D. melanogaster*, are represented. ND, not described.

Supplementary Table 2 Planarian homologs of activator and inhibitor genes whose expression is modulated in response to amputation associated with infection.
