# Supplementary material for: Planarians (Platyhelminthes)—An Emerging Model Organism for Investigating Innate Immune Mechanisms
Source: Front Cell Infect Microbiol. 2021 Mar 1;11:619081. doi: 10.3389/fcimb.2021.619081 (PMC7958881; doi:10.3389/fcimb.2021.619081)
Supplement: Supplementary Table 1 — List of the 16 bacterial species used in the Abnave et al. study (Abnave et al., 2014). The three main classes of pathogens responsible for human diseases and pathogens of model organisms, such as C. elegans or D. melanogaster, are represented. ND, not described. [file DataSheet_1.docx]

**Table S1.** Pathogens used to infect planarians by Abnave et al.

|  |  | Pathogen for : | | |
| --- | --- | --- | --- | --- |
| Class of | Species | *H. sapiens* | *C. elegans* | *D. melanogaster* |
| organism |  |  |  |  |
| Gram negative | *B. cepacia* | pneumonia and septicemia | yes | yes |
|  | *B. multivorans* | fulminant pneumonia | yes | yes |
|  | *B. melitensis* | brucellosis | ND | ND |
|  | *L. pneumophila* | pneumonia | yes | ND |
|  | *S. marcescens* | bacteremia, pneumonia | yes | yes |
|  | *S. typhimurium* | salmonellosis | yes | yes |
| Gram positive | *B. thuringiensis* | soft-tissue infection, necrosis | yes | yes |
|  | *C. jeikeium* | sepsis, endocarditis | ND | ND |
|  | *E. faecalis* | various wound infection; bacteremia | yes | yes |
|  | *L. monocytogenes* | listeriosis | yes | yes |
|  | *S. aureus* | pneumonia, abscess, sepsis, toxic shock syndrome | yes | yes |
|  | *S.pneumoniae* | pneumonia, septicemia, meningitis | yes | yes |
| Mycobacteria |  |  |  |  |
|  | *M. avium* | pulmonary infection | ND | ND |
|  | *BCG* | opportunistic infection | ND | ND |
|  | *M. marinum* | cutaneous ulcers, nodules, or nodular lymphangitis | ND | yes |
|  | *M. tuberculosis* | tuberculosis | ND | ND |
|  |  |  |  |  |

ND: Not Described

**Table S2.** homologous genes to H. sapiens or D. melanogaster reference genes categorized them in mediating (activators) or reducing (inhibitors) signal transduction

|  |  |  |
| --- | --- | --- |
|  | **genes** | |
| **Function** | activators | inhibitors |
|  | *Smed-mkk6-1* | *Smed-pp6* |
|  | *Smed-mkk4* | *Smed-ppm1b* |
|  | *Smed-p38-1* | *Smed-ppm1a* |
|  | *Smed-tak1* | *Smed-cyld-1* |
|  | *Smed-jun D* |  |
|  | *Smed-hep* |  |
|  | *Smed-Traf2* |  |
|  | *Smed-Traf2-1* |  |
|  | *Smed-jnk* |  |
|  | *Smed-tab1-1* |  |
|  | *Smed-xiap* |  |
|  | *Smed-pgrp-1* |  |
|  | *Smed-pgrp-2* |  |
|  | *Smed-pgrp-3* |  |
|  |  |  |
